# Sensitive Skin Improvement Through Bioinformatics-Identified Cosmetic Ingredients That Regulate Transcriptome-Derived Biomarkers

**DOI:** 10.3390/biom16060843

**Published:** 2026-06-09

**Authors:** Seo Hyeong Kim, Ji Hye Kim, Ji Min Shin, Yoon Mi Choi, Da Som Kim, Su Min Seo, Eun Young Jang, Sung Jae Lee, Jin-Muk Lim, Minsoo Han, Do Hyeon Jeong, Kwang Hoon Lee

**Affiliations:** 1 Cutis Biomedical Research Center Co., Ltd., Seoul 07327, Republic of Korea; shk@cutis.kr (S.H.K.); jhk@cutis.kr (J.H.K.); jms@cutis.kr (J.M.S.); ymc@cutis.kr (Y.M.C.); dsk@cutis.kr (D.S.K.); ssm@cutis.kr (S.M.S.); eyj@cutis.kr (E.Y.J.); sjlee@cutis.kr (S.J.L.); 2 Precision Medicine Institute, Innowl Co., Ltd., Seoul 07801, Republic of Korea; jmlim@innowl.io; 3 AI Institute, Alopax-Algo Co., Ltd., Seoul 07801, Republic of Korea; hms@alopaxalgo.com; 4 Raphas Co., Ltd., Seoul 07793, Republic of Korea; tony@raphas.com

**Keywords:** sensitive skin, transcriptomics, biomarker, bioinformatics, cosmeceutical, microneedle

## Abstract

Sensitive skin is characterized by hypersensitivity to normal stimuli, and objective diagnostic tools and treatments are still limited. Currently, cosmetics for sensitive skin are developed through the exclusion of known irritants rather than investigation into the underlying mechanisms of sensitivity. In this study, we developed an integrated pipeline combining transcriptome analysis via microneedle-based skin sampling (MISSM), bioinformatics, in vitro validation, and clinical assessment to identify sensitive skin-associated inflammatory biomarkers and cosmetic ingredients that regulate them. Candidate biomarkers and matched cosmetic ingredients were identified from transcriptomic data and validated in lactic acid-stimulated HaCaT and human dermal fibroblasts via qRT-PCR. A prototype emulsion was developed and evaluated in a 4-week open-label pilot clinical trial with longitudinal molecular monitoring via MISSM. After lactic acid stimulation, sensitive skin-associated biomarkers (*MCOLN1*, *CYR61*, *PMAIP1*, *PTGS2*, and *HMGB2*) were significantly upregulated in both cell types, and cosmetic ingredients that regulate these biomarkers were confirmed in vitro. The emulsion prototype demonstrated hypoallergenicity in a primary irritation test. In the pilot clinical trial, target biomarker expression was significantly reduced in MISSM-derived samples, with improvements in skin hydration, barrier function, redness, and sensory reactivity also observed. This integrated pipeline will enable the discovery of inflammatory biomarker-regulating cosmetic ingredients, with potential applicability to various inflammatory skin conditions.

## 1. Introduction

Sensitive skin refers to skin that experiences unpleasant sensations such as stinging, burning, pain, itching, and pulling not only from irritating substances but also from normal stimuli such as cosmetic ingredients or environmental changes in the absence of a skin disease [1,2,3]. Epidemiological studies suggest that approximately 44% of adults in the United States and 38% of adults in Europe have sensitive skin, and the prevalence is estimated to be increasing due to stress and a reduced quality of life [4,5,6].

Despite the growing awareness and need regarding treatment for sensitive skin, objective assessment methods, diagnostics, and therapies are still limited [7]. Currently, cosmetics for sensitive skin are being developed that exclude known irritants such as parabens, sulfates, and fragrance allergens [8]. However, these formulations do not target the underlying molecular pathophysiological mechanisms, leaving the biological basis of skin sensitivity poorly addressed.

Previous studies have suggested that upregulated transient receptor potential (TRP) channels, inflammatory reactions, epidermal barrier disruption, and microvascular hypersensitivity leading to persistent erythema are the main causes of sensitive skin [9,10,11,12]. Molecular biological analysis using actual sensitive skin samples is crucial in understanding these sensitive skin mechanisms. However, currently, the commonly used skin biopsy method for obtaining skin specimens is highly invasive, resulting in low patient compliance and requiring surgeons’ expertise. To address these limitations, we utilized a minimally invasive skin sampling method using microneedle patches (MISSM), which we developed in our previous study, to collect skin samples with minimal discomfort [13].

Recently, studies have been conducted to identify sensitive skin-associated biomarkers and novel candidate regulatory factors through bioinformatics analysis profiling the transcriptome of sensitive skin [14,15,16]. However, platforms that integrate a whole cycle of stimulation and efficacy evaluation through bioinformatics analysis and the derivation of biomarkers and candidate factors, in vitro efficacy verification, prototype development and stability evaluation, and further clinical trials are not yet common. To this end, we conducted MISSM-based transcriptome analysis for sensitive skin, bioinformatics-based biomarker and cosmetic ingredient discovery, in vitro efficacy testing in an induced sensitive skin cell model, prototype development, and a pilot clinical trial targeting sensitive skin.

Therefore, the primary goals in this study were to identify sensitive skin-associated biomarkers through transcriptomics and bioinformatics, to validate cosmetic ingredients that regulate these biomarkers in vitro and in vivo, and to provide molecular evidence supporting a mechanism-based approach to sensitive skin research.

## 2. Materials and Methods

### 2.1. Bioinformatic Analysis

We conducted a bioinformatic analysis using previously published transcriptome data (GSE179925, available at NCBI GEO) on five participants with sensitive skin of the stinging type and five participants with non-sensitive skin. To identify candidate biomarkers specific to sensitive skin, we performed differential gene expression analysis, principal component analysis (PCA), gene set enrichment analysis (GSEA), Gene Ontology biological process (GO_BP), Reactome, and Kyoto Encyclopedia of Genes and Genomes (KEGG) pathway analyses. We also explored cosmetic ingredients that could regulate the identified biomarkers.

#### Biomarker–Compound Matching Analysis

To identify cosmetic ingredients capable of regulating sensitive skin-associated biomarkers, a biomarker–compound matching analysis was performed using the Comparative Toxicogenomics Database (CTD; https://ctdbase.org/ (accessed on 29 May 2024)), a publicly available database integrating chemical–gene interactions, functional annotations, and literature-curated toxicogenomic relationships.

First, candidate biomarkers identified through transcriptomic and pathway analyses were individually queried in the CTD. For each biomarker, the “Chemical Interactions” dataset was examined for downstream filtering. Chemical compounds were subsequently screened based on interaction directionality, focusing on compounds reported to “decrease expression” of upregulated sensitive skin-associated biomarkers or “increase expression” of downregulated biomarkers.

Next, candidate compounds were filtered according to cosmetic applicability, including topical usability, previously reported cosmetic or dermatological use, safety profile, formulation feasibility, and natural origin preference when applicable. Compounds considered unsuitable for cosmetic application due to toxicity, regulatory restrictions, or poor safety profiles were excluded.

Finally, PubMed literature searches were conducted to validate previously reported relationships between the selected compounds and target biomarker regulation. Based on this multi-step filtering process, Schizandrin B, genistein, folic acid, allantoin, and betaine were selected as candidate cosmetic ingredients for experimental validation.

### 2.2. In Vitro Analysis

#### 2.2.1. Ingredients

Schizandrin B (PHL89786; Sigma-Aldrich, St. Louis, MO, USA), schisandra (CH Schisandra Chinensis Fruit Extract; CH Harmony Co., Ltd., Anyang, Republic of Korea), genistein (Ecotechinc Co., Ltd., Bucheon, Republic of Korea), folic acid (Cosroma^®^ VB9; Shanghai Cosroma Biotech Co., Ltd., Shanghai, China), allantoin (Cnkinternational Co., Ltd., Busan, Republic of Korea), and betaine (Cnkinternational Co., Ltd., Busan, Republic of Korea) were used.

#### 2.2.2. Cell Culture

HaCaT cells were cultured in high-glucose Dulbecco’s modified Eagle’s medium (DMEM; HyClone, Logan, UT, USA) supplemented with 10% fetal bovine serum (FBS; Gibco, Grand Island, NY, USA) and 1% penicillin/streptomycin (Lonza, Basel, Switzerland). Human dermal fibroblasts (HDFs) were cultured in RPMI-1640 medium (Hyclone, Logan, UT, USA) supplemented with 10% FBS and 1% penicillin/streptomycin. The cells were maintained in a humidified atmosphere at 37 °C and 5% CO_2_ using a water-jacketed CO_2_ incubator (Thermo Fisher Scientific, Waltham, MA, USA). The cells were cultured in 100 mm dishes. The growth medium was changed every 24–48 h, depending on cell confluency.

#### 2.2.3. Cell Toxicity Test

The Cell Proliferation Kit I (Roche, Basel, Switzerland) was used to evaluate the cytotoxicity of the ingredients at various concentrations. Cells were seeded in 24-well plates at 1 × 10^5^/well and incubated for 24 h. After 24 h, the medium was replaced with serum- and additive-free DMEM or RPMI-1640, and the ingredients were added at the relevant experimental concentrations. After another 24 h, the medium was changed to a medium containing 0.5 mg/mL 3-(4,5-dimethylthiazol-2-yl)-2,5-diphenyltetrazolium bromide (MTT) and incubated for at least 4 h. After the incubation period, the medium was aspirated, and 300 µL of dimethyl sulfoxide (DMSO; Daejung Chemicals & Metals Co., Ltd., Siheung, Republic of Korea) was added to each experimental and control well to dissolve the formazan crystals that formed during incubation. The solutions were transferred to 96-well plates, and the absorbance was measured at 585 nm.

Cell viability was consequently calculated using the following equation:

% Cell viability = (OD_Sample_ − OD_Blank_)/(OD_Control_ − OD_Blank_) × 100

where

• OD_Sample_ = mean absorbance of treated cells;
• OD_Blank_ = mean absorbance of blank (DMSO);
• OD_Control_ = mean absorbance of untreated control cells.

#### 2.2.4. In Vitro Efficacy Test

Cells were seeded at a density of 1 × 10^5^ cells/well in 24-well plates and incubated for 24 h. After 24 h, the medium was replaced with serum and additive-free DMEM (for HaCaT cells) or RPMI-1640 (for HDFs). The cells were stimulated with lactic acid (HaCaT: 0.04%, HDF: 0.01%) and treated with the candidate ingredients for 24 h. After 24 h of incubation, the medium was aspirated, and the cells were suspended by treatment with 0.05% trypsin/EDTA (Gibco, Grand Island, NY, USA). Total RNA was extracted using the XENOPURE-Total RNA Purification Kit (Xenohelix, Incheon, Republic of Korea), and cDNA was synthesized using the XENO cDNA synthesis kit (Xenohelix, Incheon, Republic of Korea) using a Veriti thermal cycler (Applied Biosystems, Foster City, CA, USA). Quantitative real-time polymerase chain reaction (qRT-PCR) was performed at least twice for each sample using cDNA supplemented with the appropriate primers for *MCOLN1* (Hs01100653_m1; Thermo Fisher Scientific, Waltham, MA, USA), *CYR61 *(CUTIS-Ss-U2-M1; Xenohelix, Incheon, Republic of Korea), *PMAIP1 *(CUTIS-Ss-U2-M4; Xenohelix, Incheon, Republic of Korea), *PTGS2 *(CUTIS-Ss-U2-M3; Xenohelix, Incheon, Republic of Korea), and *HMGB2 *(CUTIS-Ss-U2-M5; Xenohelix, Incheon, Republic of Korea), and *ACTIN* (CUTIS-Ct-ACTIN; Xenohelix, Incheon, Republic of Korea) with a LineGene qPCR system (BIOER, Hangzhou, China). The relative quantification was performed using the following equation:

Relative quantification (RQ) = 2−ΔΔC_T_

ΔΔC_T_ = ΔC_T_ (Experimental) − ΔC_T_ (Control)

ΔC_T_ = C_T_ (Target gene) − C_T_ (Housekeeping gene)

where

• Experimental = lactic acid and/or ingredient-treated cells;
• Control = untreated control cells;
• Target gene = *MCOLN*, *CYR61*, *PMAIP1*, *PTGS2*, and *HMGB2*;
• Housekeeping gene = *ACTIN*.

### 2.3. Formulation and Stability Assessment of the Prototype Emulsion

The prototype formulation was an oil-in-water (O/W) lotion-type emulsion manufactured by Greencos Co., Ltd. (Gimpo, Republic of Korea) in accordance with the manufacturer’s standard cosmetic emulsification process. Briefly, the aqueous and oil phases were prepared and heated separately, then combined under homo-mixer emulsification in a main reactor tank, followed by controlled cooling with continued mixing, defoaming, and filtration prior to filling. The formulation incorporated key active ingredients including Schisandra chinensis extract, genistein, allantoin, folic acid, and betaine. Physicochemical characterization confirmed a homogeneous white lotion appearance with a pH of 6.70 ± 1.00, a viscosity of 4200 ± 800 cps as determined by a Brookfield RVDV-E viscometer (Brookfield, Middleboro, MA, USA; spindle No. 6, 50 rpm, 60 s), and a specific gravity of 1.00–1.04, consistent with the properties of a stable conventional O/W cosmetic emulsion.

The stability of the test products was evaluated under three different conditions (Table 1): the temperature variation condition, 14 cycles of cooling–heating treatment for 2 weeks; the accelerated long-term condition, 40 ± 2 °C and 75 ± 5% R.H. for 3 months; and the general long-term condition, 25 ± 2 °C and 60 ± 5% R.H. for 3 months. The appearance, odor, pH, and viscosity of the test products were evaluated at each time point. Appearance was measured using eye perception, and odor was measured using nose perception. pH was measured using a pH meter (Orion Star A211; Thermo Fisher Scientific, Waltham, MA, USA). The viscosity was measured using a viscometer (DV2TLV, Brookfield, Middleboro, MA, USA) and is expressed in millipascal second (mPa·s) units.

### 2.4. Hypoallergenicity Assessment in Participants with Sensitive Skin

To confirm the safety of the prototype emulsion prior to the efficacy trial, hypoallergenicity was assessed using the IQ chamber patch test in 33 participants with stinging-type sensitive skin. Participants were selected using the lactic acid stinging test based on the inclusion and exclusion criteria, and written informed consent was obtained from each participant. Demographic details, including the average age and sex of the participants, are presented in Table 2. An IQ chamber loaded with 20 μL of test product was placed on the participants’ backs, covered with an occlusive patch, and applied for 24 h. After 24 h, the patch was removed, and skin reactions were assessed 1 h and 24 h after patch removal. The skin reaction value was determined based on the International Contact Dermatitis Research Group grading system as follows: −(0, no reaction), ±(0.5, doubtful reaction), +(1, weak positive reaction), ++(2, strong positive reaction), +++(3, extreme positive reaction). The mean skin reaction score was classified as follows: 0.0–0.9 (low irritation), 1.0–2.9 (mild irritation), 3.0–4.9 (moderate irritation), and >5.0 (strong irritation) [17].

### 2.5. Pilot Clinical Trial: Molecular and Physiological Efficacy Assessment

#### 2.5.1. Participants

To evaluate both the molecular and physiological effects of the prototype emulsion, a pilot clinical trial was conducted. This study was approved by the Cutis Institutional Review Board (approval number: CTS-IRB-24O17-I19; approved on 7 October 2024). A total of 23 participants were enrolled in the study between September 2024 and October 2024. Participants were selected based on specific inclusion and exclusion criteria, and written informed consent was obtained from each participant prior to their participation. All experiments involving human participants were conducted in accordance with the principles outlined in the Declaration of Helsinki. Demographic details, including the average age and sex of the participants, are presented in Table S1. Following facial cleansing, participants were acclimated for 30 min in an examination room maintained at 22 ± 2 °C with a relative humidity (R.H.) of 40–60% prior to further procedures. The participants applied the test product to their faces twice a day, in the morning and evening, for 4 weeks. Measurements were taken by visiting the center before, 2 weeks after, and 4 weeks after using the test product.

#### 2.5.2. Non-Invasive Devices

Skin-soothing effect measurements were performed on the front, right, and left sides of the participants’ faces using VISIA-CR (Canfield Scientific Inc., Parsippany, NJ, USA). VISIA-CR is a high-resolution photography device that has six built-in flashes and captures images with a combination of lights. The cheek area of the face was designated using I-MAX PLUS (INGPLUS Co., Ltd., Seoul, Republic of Korea), an image analysis program for images captured with VISIA-CR, and the a* value (redness) was analyzed within the area. As the redness of the skin decreased, the a* value decreased, indicating that the redness had been soothed.

The Corneometer^®^ CM 825 (Courage and Khazaka Electronic GmbH, Cologne, Germany) assessed skin surface hydration at a depth of 10–20 μm within the stratum corneum. The value of the Corneometer (ε) is correlated with the skin moisture level.

Transepidermal water loss (TEWL) was evaluated using the Tewameter^®^ TM 300 (Courage and Khazaka Electronic GmbH, Cologne, Germany), which indicates skin barrier function, with higher TEWL values signifying barrier impairment.

#### 2.5.3. Lactic Acid Stinging Test

A lactic acid stinging test (LAST) was conducted following a previously described protocol with slight modifications [18]. A cotton swab soaked in a 10% lactic acid solution was applied to the left nasolabial fold, while a cotton swab soaked in a saline solution was applied to the right nasolabial fold. Participants rated the intensity of sensations, including stinging, itching, or burning, using a 4-point scale (0 = none, 1 = slight, 2 = moderate, and 3 = severe) at intervals of 0, 2.5, 5, 8, and 10 min post-application. After 10 min, the area was rinsed with water. The stinging score was calculated using the following formula: score = (sum of the lactic acid score − sum of the saline solution score)/12.

#### 2.5.4. Skin Sampling with Microneedle Patches

For longitudinal molecular monitoring, skin samples were acquired using biocompatible microneedle patches (Raphas, Seoul, Republic of Korea). Sampling was conducted before treatment and at 2 and 4 weeks after application to the nasolabial fold lesion. The collected skin samples were stored individually at room temperature in 1.5 mL tubes containing lysis buffer until further use. No adverse effects were observed following patch application.

#### 2.5.5. Total RNA Extraction and qRT-PCR

Using microneedle patches soaked in lysis buffer from the XENOPURE-Total RNA Purification Kit (Xenohelix, Incheon, Republic of Korea), subsequent steps were performed as described in Section 2.2.4 In vitro efficacy test.

As the cycle threshold (C_T_) value varied depending on the amount of cDNA used in the reaction, it was normalized to the C_T_ value of the housekeeping gene actin. The ΔC_T_ value, obtained by subtracting the C_T_ value of actin from the C_T_ value of the target gene (*MCOLN1*, *CYR61*, *PMAIP1*, *PTGS2*, and *HMGB2*), was used for the analysis. A decrease in the ΔC_T_ value indicated a corresponding increase in target gene expression.

### 2.6. Statistical Analysis

Statistical significance was set at *p* < 0.05, with a 95% confidence interval, using the IBM SPSS Statistics (version 27, IBM Corp., Armonk, NY, USA). For the in vitro efficacy test analysis, data were analyzed using one-way analysis of variance (ANOVA), and the significance of the differences was determined using the Tukey–Kramer test. For the clinical test, repeated-measures ANOVA was used as a parametric method after the normality test for data was carried out three or more times. Post hoc analysis was performed using the Bonferroni method. For nonparametric testing, the Friedman test was used, and paired comparisons were performed using the Wilcoxon signed-rank test with Bonferroni correction applied to assess significance post hoc.

## 3. Results

### 3.1. Bioinformatic Analysis with Transcriptomics of Participants with Sensitive Skin

**Figure 1:**
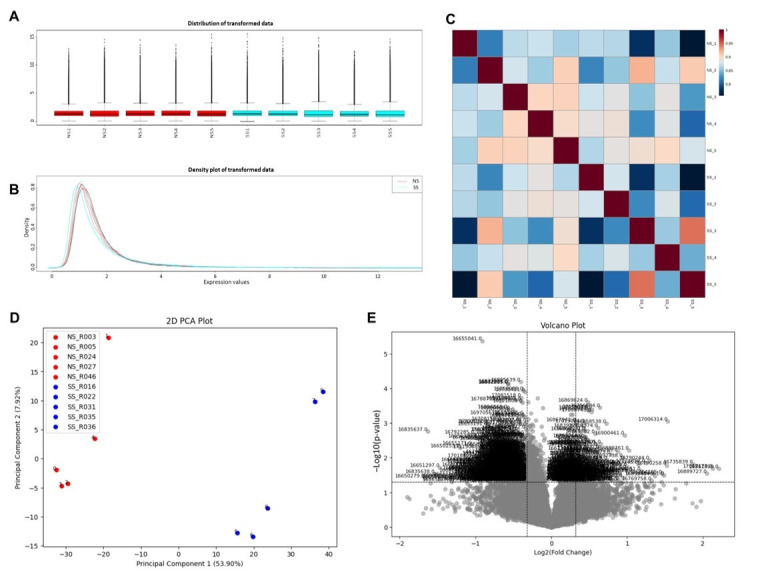
Data quality and basic analysis of transcriptomic data. The quality of transcriptomic data was analyzed using (**A**) a box plot, (**B**) a density plot, and (**C**) Pearson’s correlation. Through (**D**) PCA and (**E**) volcano plot analysis, the difference in gene expression between sensitive skin and non-sensitive skin was visualized.

Data quality was assessed by analyzing the skin microarray data of participants with sensitive and non-sensitive skin. A box plot was used to compare expression levels and check the consistency of samples (Figure 1A), and a density plot, generated through kernel density estimation, visualized the overall data distribution (Figure 1B). Expression levels were consistent across all samples, with no identified outliers. Pearson’s correlation, used to evaluate the similarity or relevance between samples, confirmed that there was no significant correlation between the two groups across all probes (Figure 1C). Subsequently, PCA was performed to reduce the number of dimensions by expressing the multidimensional data with a small number of main components. Probes with *p* < 0.05 were selected and analyzed, confirming that the sensitive skin group and the non-sensitive skin group were clearly separated (Figure 1D). In addition, the presence of various statistically different gene groups between the two groups was confirmed through a volcano plot, an analysis method used to find genes that satisfy both fold change and *p*-value (Figure 1E).

**Figure 2:**
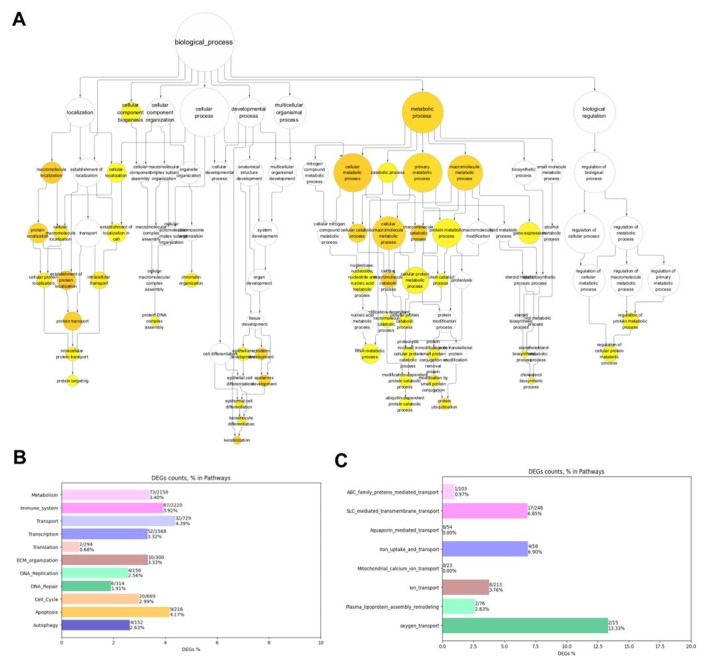
Pathway analysis of transcriptomic data. (**A**) The GO_BP analysis of transcriptomic data of sensitive skin. The number of genes significantly different for (**B**) each major mechanism, especially (**C**) the transport pathway.

Next, a mechanistic analysis was performed. GO_BP pathway analysis confirmed that metabolic processes and transport were activated (Figure 2A), and the number of genes significantly different for each major mechanism is shown in a graph (Figure 2B). Additional analyses were performed with a focus on the transport pathway (Figure 2C). Together with *CYR61*, *PMAIP1*, *PTGS2*, and *HMGB2*, previously discovered in our earlier research [19], *MCOLN1* showed increased expression in sensitive skin. *MCOLN1* is a protein involved in the SLC-mediated transmembrane transport pathway and functions as a component of the TRPs responsible for Ca2+ transport [20].

Subsequently, we performed a literature-guided biomarker–compound matching analysis using the Comparative Toxicogenomics Database (CTD) to identify candidate cosmetic ingredients capable of modulating the identified biomarkers. Candidate compounds were prioritized based on reported gene expression regulatory effects, interaction directionality, cosmetic applicability, topical safety, and formulation feasibility. The selected compounds were further validated through a PubMed-based literature review to confirm previously reported associations with biomarker regulation. Through this process, Schizandrin B, genistein, folic acid, allantoin, and betaine were selected as candidate cosmetic ingredients targeting *MCOLN1*, *CYR61*, *PMAIP1*, *PTGS2*, and *HMGB2*, respectively (Table 3).

### 3.2. Verification of Cosmetic Ingredient Candidates That Regulate Sensitive Skin Biomarkers

**Figure 3:**
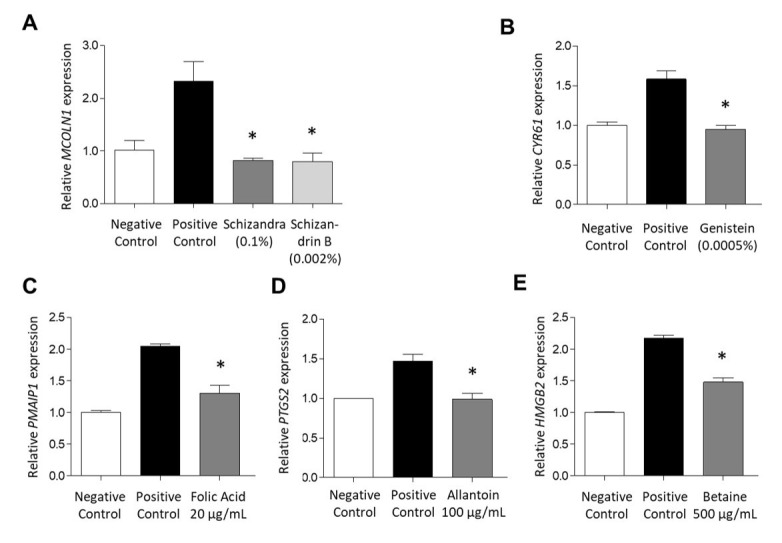
In vitro analysis of candidates in HaCaT cells. Quantitative real-time PCR analyses of (**A**) *MCOLN1*, (**B**) *CYR61*, (**C**) *PMAIP1*, (**D**) *PTGS2*, and (**E**) *HMGB2* in negative control, positive control (lactic acid-treated), and candidate cosmetic ingredient-treated groups. Data are presented as mean ± standard error of the mean. * *p* < 0.05 vs. positive control.

**Figure 4:**
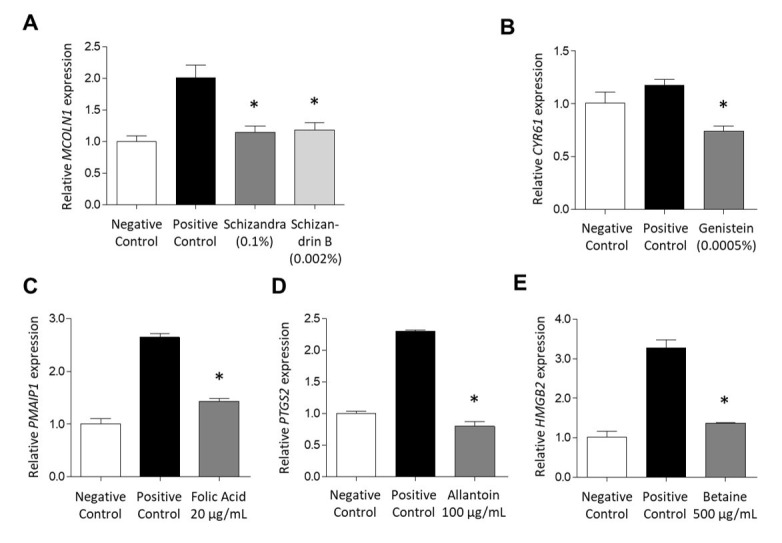
In vitro analysis of candidates in human dermal fibroblasts (HDFs). Quantitative real-time PCR analyses of (**A**) *MCOLN1*, (**B**) *CYR61*, (**C**) *PMAIP1*, (**D**) *PTGS2*, and (**E**) *HMGB2* in negative control, positive control (lactic acid-treated), and candidate cosmetic ingredient-treated groups. Data are presented as mean ± standard error of the mean. * *p* < 0.05 vs. positive control.

We attempted to verify whether the cosmetic ingredient candidates discovered through biomarker–compound matching analysis could regulate the biomarker in vitro. Two types of skin cells (HaCaTs and HDFs) were used, and the appropriate treatment concentration for each ingredient was determined using an MTT assay (Figures S1 and S2). Next, HaCaT and HDF cells were treated with appropriate concentrations of the ingredients to confirm target biomarker expression via qRT-PCR. To simulate a sensitive skin-associated environment, a lactic acid-treated group was used as a positive control. In the case of Schizandrin B, which regulates *MCOLN1*, there is no cosmetic-grade material; therefore, its efficacy was additionally verified in Schisandra, which contains some Schizandrin B. When compared to the positive control group (stimulated with lactic acid), schisandra (0.1%) and Schizandrin B (0.002%) inhibited the expression of increased *MCOLN1* (Figure 3A), genistein (0.0005%) inhibited the expression of increased *CYR61* (Figure 3B), folic acid (20 μg/mL) inhibited the expression of increased *PMAIP1* (Figure 3C), allantoin (100 μg/mL) decreased the expression of increased *PTGS2* (Figure 3D), and betaine (500 μg/mL) decreased the expression of increased *HMGB2* (Figure 3E) significantly. These results were identified not only in HaCaT but also in HDF cells (Figure 4A–E).

### 3.3. Formulation and Stability Verification of the Biomarker-Targeting Emulsion

**Figure 5:**
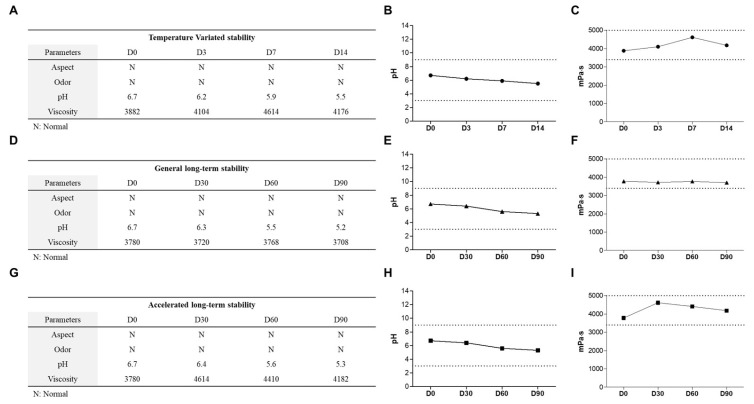
Stability testing of emulsion containing biomarker-targeting ingredients. The physicochemical stability of the test emulsion was evaluated under three conditions: short-term temperature cycling (**A**–**C**), long-term storage at ambient temperature (**D**–**F**), and accelerated storage (**G**–**I**). Assessment included (**A**,**D**,**G**) appearance and odor, (**B**,**E**,**H**) pH (target: 3–9), and (**C**,**F**,**I**) viscosity (target: 3500–5000 mPa·s). No abnormal changes were observed across all conditions, indicating robust product stability.

Having confirmed the biomarker-regulatory activity of the candidate ingredients in vitro, we formulated a prototype emulsion incorporating these bioinformatically identified components and verified its physicochemical stability prior to clinical evaluation. Before we evaluated the efficacy of the manufactured test product, its stability was assessed under three conditions (Table 1). The parameters measured were appearance, odor, pH, and viscosity. For 2 weeks in the temperature-varied stability test, appearance and odor were observed to be normal (Figure 5A), pH measured within the normal range of 3 to 9 (Figure 5A,B), and viscosity measured in the normal range, between 3500 and 5000 (Figure 5A,C). For 3 months in the general long-term stability test, appearance and odor were observed to be normal (Figure 5D). pH measured within the normal range of 3 to 9 (Figure 5D,E), and viscosity measured within the normal range of 3500 to 5000 (Figure 5D,F). For 3 months in the accelerated long-term stability test, appearance and odor were also observed to be normal (Figure 5G), pH measured within the normal range of 3 to 9 (Figure 5G,H), and viscosity measured within the normal range of 3500 to 5000 (Figure 5G,I). Through these tests, we confirmed the stability of the test product under varying temperatures and during long-term storage.

### 3.4. Hypoallergenicity Assessment of the Prototype Emulsion in Participants with Sensitive Skin

Prior to the efficacy trial, the hypoallergenicity of the prototype emulsion was confirmed in participants with stinging-type sensitive skin using an IQ chamber patch test. A total of 33 participants attached an IQ chamber loaded with 20 μL of the test product to their backs for 24 h, after which it was removed, and the skin response was evaluated after 1 h and 24 h. No participants had a skin response; thus, the skin irritation score of the test product was 0.00 (Table 4A,B).

### 3.5. Clinical Efficacy Test

An open-label, 4-week pilot clinical trial was conducted to evaluate the molecular and physiological effects of the prototype emulsion in participants with sensitive skin, with longitudinal biomarker monitoring via MISSM as the primary molecular endpoint. A total of 23 female participants with sensitive skin were enrolled in the study. All participants voluntarily provided informed consent, and their demographic details are summarized in Table S1. The participants were instructed to apply the test product to their entire face twice daily, in the morning and evening. Non-invasive skin assessment devices (VISIA-CR, Corneometer, Tewameter), the Lactic Acid Sting Test, and MISSM were used at week 0 (before application), 2, and 4 after application to evaluate the efficacy of the test product.

**Figure 6:**
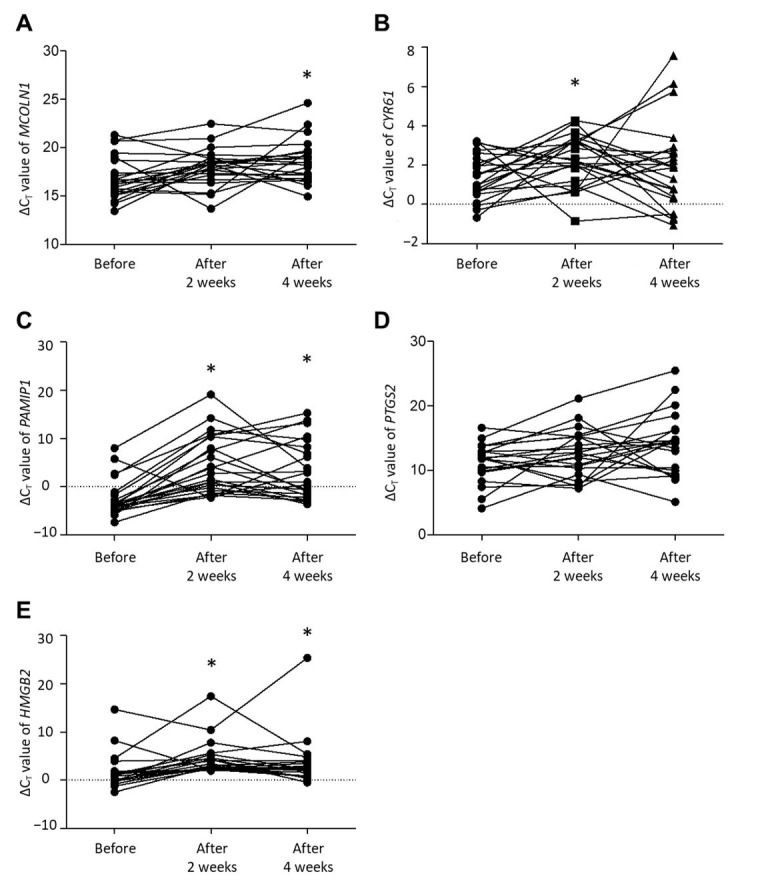
In vivo regulation of sensitive skin biomarkers following 4-week product application. ΔC_T_ values of five biomarkers from MISSM-derived skin samples were measured via qRT-PCR at baseline, week 2, and week 4. (**A**) *MCOLN1*, (**B**) *CYR61*, (**C**) *PMAIP1*, (**D**) *PTGS2*, and (**E**) *HMGB2* all showed expression normalization trends post-treatment, with statistically significant downregulation of molecular markers linked to sensitive skin pathophysiology. * *p* < 0.05 vs. baseline.

Molecular outcomes*:* To assess the primary molecular endpoint, biomarker expression in MISSM-derived skin samples was monitored longitudinally via qRT-PCR (Table 5 and Figure 6). As a higher ΔC_T_ value indicates lower target gene expression, an increase in ΔC_T_ reflects the downregulation of the corresponding biomarker. ΔC_T_ of *MCOLN1* showed a tendency to increase 1.104 cycles (*p* = 0.114) at week 2 and a statistically significant increase of 1.782 cycles (*p* = 0.010) at week 4. ΔC_T_ of *CYR61* showed a statistically significant increase of 0.871 cycles (*p* = 0.016) at week 2 and a tendency to increase 0.543 cycles (*p* = 0.330) at week 4. *PMAIP1* showed statistically significant changes in ΔC_T_ by 7.011 cycles (*p* < 0.001) in week 2 and by 5.786 cycles (*p* < 0.001) in week 4, all of which were statistically significant changes up to week 4. ΔC_T_ of *PTGS2* showed a tendency to increase by 1.397 cycles (*p* = 0.224) in week 2 and by 2.756 cycles (*p* = 0.078) in week 4, but the difference was not statistically significant. ΔC_T_ of *HMGB2* increased by 2.737 cycles (*p* = 0.004) at week 2 and by 1.911 cycles (*p* = 0.005) at week 4, showing statistically significant changes in all cases up to week 4. These results demonstrate that applying the product twice daily for 4 weeks resulted in robust phenotypic and molecular improvements in sensitive skin, supporting its mechanism-based efficacy.

**Figure 7:**
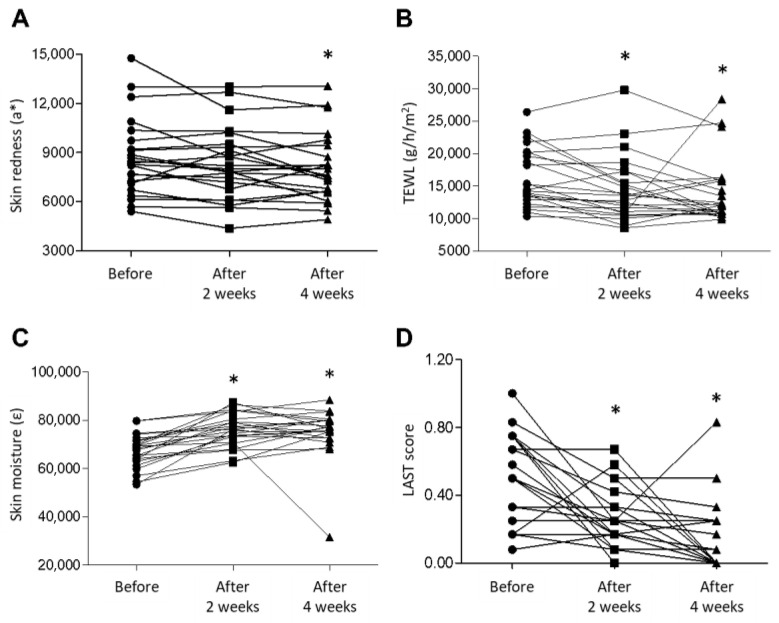
Clinical improvements in biophysical and sensory symptoms after 4 weeks of product use. An open-label clinical trial was conducted in participants with sensitive skin. Non-invasive measurements showed (**A**) a reduction in erythema (a* value), (**B**) a decrease in transepidermal water loss (TEWL), (**C**) increased stratum corneum hydration, and (**D**) a reduction in Lactic Acid Sting Test (LAST) scores from baseline to weeks 2 and 4. Data are presented as mean ± SD. * *p* < 0.05 vs. baseline.

Physiological Outcomes*:* In addition to molecular changes, non-invasive measurements confirmed physiological improvements after topical application (Table 6 and Figure 7). Skin redness (a* value) decreased from 8.713 ± 2.352 at week 0 to 8.360 ± 2.216 at week 2 (*p* = 0.339) and to 8.102 ± 2.110 at week 4 (*p* = 0.01) (Figure 7A). TEWL decreased from 16.275 ± 4.498 g/m^2^/h to 14.482 ± 5.015 g/m^2^/h at week 2 (*p* = 0.009) and to 14.121 ± 5.066 g/m^2^/h at week 4 (*p* = 0.018) (Figure 7B). Hydration increased from 67.217 ± 7.090 to 75.832 ± 6.836 at week 2 (*p* < 0.001) and to 75.038 ± 10.604 at week 4 (*p* < 0.001) (Figure 7C). LAST scores decreased from 0.518 ± 0.266 to 0.257 ± 0.178 at week 2 (*p* = 0.002) and to 0.137 ± 0.206 at week 4 (*p* < 0.001) (Figure 7D). These results confirmed statistically and clinically significant reductions in erythema, the restoration of barrier function, an improvement in hydration, and a reduction in hypersensitivity.

Table 7 summarizes the biomarker-based cosmetic ingredient discovery and validation pipeline in this study, including in vitro and clinical efficacy outcomes.

## 4. Discussion

Sensitive skin is clinically classified into acneiform, rosacea-like, stinging, and allergic skin types, and the pathophysiological mechanisms associated with each phenotype are known to include barrier damage, neurosensory hyperexcitability, immune dysregulation, and microvascular reactivity [27]. Currently, most cosmetics for sensitive skin are marketed without the addition of known irritants such as surfactants, fragrance allergens, and preservatives. While this may temporarily alleviate symptoms, it fails to address the underlying mechanisms that cause sensitivity in skin [28]. In particular, the stinging type mediated by C-fiber activation and TRP channels is unlikely to be substantially improved by avoiding the addition of irritants [29].

To address these limitations, we devised a cosmeceutical development pipeline that regulates molecular biological mechanisms for improving sensitive skin using our MISSM-based biomarker discovery and efficacy evaluation platform [19,30]. This pipeline integrates transcriptome profiling of MISSM samples, bioinformatics analysis to identify biomarkers and their regulatory cosmetic ingredients, in vitro validation, prototype development, and a pilot clinical trial. A 4-week clinical trial confirmed that the modulation of sensitive skin-specific biomarkers resulted in clinically significant improvements in skin barrier function, erythema, hydration, and sensory reactivity.

Transcriptome analysis of MISSM samples from sensitive skin identified the specific expression of genes involved in ion transport, inflammatory signaling, and skin barrier dysfunction. In particular, *MCOLN1* was identified as a novel, sensitive skin-associated biomarker within the TRP channel family, complementing previously identified sensitive skin biomarkers (*CYR61, PMAIP1, PTGS2*, and *HMGB2*) [19]. The upregulation of *MCOLN1* in sensitive skin is consistent with the involvement of abnormal Ca^2+^ flux in increased neurosensory excitability, suggesting that targeting and regulating *MCOLN1* may represent an approach to alleviating irritation-induced discomfort [31,32].

In addition, we confirmed that cosmetic ingredients discovered through bioinformatics analysis significantly regulate sensitive skin-specific biomarkers in an in vitro model that mimics a sensitive environment by inducing lactic acid. Schisandra chinensis extract and Schizandrin B significantly inhibited the expression of *MCOLN1*, genistein attenuated *CYR61*, folic acid downregulated *PMAIP1*, allantoin reduced *PTGS2*, and betaine inhibited *HMGB2*. *MCOLN1* encodes the lysosomal *TRPML1* channel, and its overexpression under oxidative stress conditions leads to excessive release of Ca^2+^ into the cytoplasm, which exacerbates neuroinflammation and mast cell degranulation [20,33,34,35]. Schizandrin B and Schisandra chinensis extract suppressed *MCOLN1* expression in vitro, suggesting a potential role in regulating intracellular Ca^2+^ signaling and attenuating C-fiber-mediated sensitization. *CYR61* acts as a matricellular amplifier of keratinocyte-derived IL-1β [36], inducing microvascular hypersensitivity [37,38,39]. Genistein, which suppressed *CYR61* expression in vitro, may contribute to reducing erythema and vascular reactivity. *PMAIP1* (NOXA) regulates stress-induced keratinocyte apoptosis [40], and its regulatory component, folic acid, regulates *PMAIP1* expression to maintain epidermal integrity. Allantoin partially inhibits *PTGS2* (*COX-2*) and thus suppresses prostaglandin-mediated vasodilation [41,42]. Betaine regulates *HMGB2* expression, thereby attenuating the TLR4 signaling cascade associated with damage and suppressing the amplification of inflammation [43,44].

The prototype containing the discovered cosmetic ingredient was tested for stability through stress tests, accelerated tests, and long-term storage tests and was confirmed to be hypoallergenic in an irritation test on participants with stinging-type skin. In addition, in a 4-week clinical trial, erythema was statistically significantly reduced only at week 4, while barrier function, hydration, and hypersensitivity (LAST score) significantly improved from week 2 to week 4. *MCOLN1* decreased to a statistically significant extent only at week 4, suggesting that improvements in gene expression require time. *CYR61*, *PMAIP1*, and *HMGB2* decreased significantly from week 2 onward, suggesting an immediate role in the initial response. Meanwhile, *PTGS2* did not show statistically significant changes, suggesting that higher doses or a longer treatment period may be necessary to fully inhibit the prostaglandin pathway.

In this study, we overcome the limitations of skin biopsy, improve participant compliance, and utilize MISSM for longitudinal sampling. The integrated pipeline presented in this study offers a novel approach that regulates underlying pathophysiological mechanisms, unlike existing approaches that simply exclude irritants. This approach is expected to be widely applicable to other sensitive skin phenotypes, such as rosacea and allergic skin, as well as skin conditions such as aging, hyperpigmentation, and atopic dermatitis.

This study has limitations, including its open-label design, relatively small sample size, and lack of a placebo control, making it difficult to completely rule out the possibility of spontaneous improvement. Therefore, future studies using a randomized, placebo-controlled design in a larger cohort are needed to evaluate long-term outcomes. Additionally, the formulation used in this study was a conventional oil-in-water (O/W) cosmetic emulsion and was not designed as a specific nano-delivery or encapsulation system. Accordingly, particle size distribution analysis and encapsulation characterization were not within the scope of the present study and represent limitations to be addressed in future work.

## 5. Conclusions

In this study, we developed an integrated pipeline encompassing MISSM-based transcriptomics and bioinformatics analysis to identify sensitive skin-associated biomarkers and cosmetic ingredients that regulate them, in vitro validation, and a pilot clinical trial. Statistically significant downregulation of *MCOLN1*, *CYR61*, *PMAIP1*, and *HMGB2* was observed following product application, accompanied by significant improvements in skin barrier function, hydration, erythema, and sensory reactivity. This pipeline is expected to provide a scalable framework for mechanism-based sensitive skin research, with potential applicability to other inflammatory skin conditions.

## 6. Patents

S.H.K., J.H.K., S.J.L., D.H.J. and K.H.L. hold patents related to the technology described in this study: Korean Patent Registration No. 10-2216945 and International Patent Application No. PCT/KR2021/009297.

## Figures and Tables

**Table 1:** Physicochemical stability test conditions and evaluation criteria for the prototype emulsion.

Test Type	Storage Condition	
	Temperature	Humidity
Temperature Variations	14 Cycle 1 Cycle (24 h): −15 °C, 8 h → 25 °C, 8 h → 45 °C, 8 h	-
General	25 ± 2 °C	60 ± 5% R.H.
Accelerated	40 ± 2 °C	75 ± 5% R.H.

Experimental conditions for stability assessment of the test product. Stability was evaluated under three storage conditions: temperature variation (14 cycles over 2 weeks), general long-term storage (25 ± 2 °C, 60 ± 5% RH), and accelerated storage (40 ± 2 °C, 75 ± 5% RH) over 3 months. Parameters assessed included appearance, odor, pH, and viscosity.

**Table 2:** Participant demographics and test protocol overview.

	Subjects
Number (*n*)	33
Sex	
Male	0
Female	33
Age (year)	51.45 ± 5.30

Summary of test population characteristics and application method. All subjects completed the 24 h occlusive patch test without protocol deviation.

**Table 3:** Cosmetic ingredient candidates targeting sensitive skin-associated biomarkers.

Candidates	Target Biomarker	PMID
Schizandrin B	*MCOLN1*	31150632 [21]
Genistein	*CYR61*	22228119 [22] 20884965 [23]
Folic Acid	*PMAIP1*	25629700 [24]
Allantoin	*PTGS2*	30654793 [25]
Betaine	*HMGB2*	26391144 [26]

Ingredient candidates identified through biomarker–compound matching analysis. Each compound was selected based on its potential to modulate a specific biomarker implicated in sensitive skin pathophysiology.

**Table 4:** Skin irritation assessment results of the test product. (**A**) Number of respondents. (**B**) Skin reaction scores.

	A									
	At 1 h After Removal of Patch					At 24 h After Removal of Patch				
	0.5	1	2	3	No. of Respondes	0.5	1	2	3	No. of Respondes
Test product	-	-	-	-	0	-	-	-	-	0
	B									
	Skin Reaction Score					Average Skin Reaction Score				
	After 1 h		After 24 h							
Test product	0.00		0.00			0.00				

(**A**) The number of respondents was assessed 1 h and 24 h after patch removal using the International Contact Dermatitis Research Group (ICDRG) grading scale. No visible irritation or erythema was observed in any subject. (**B**) Evaluation of skin reaction scores via test product patch test. All participants had a score of 0, confirming that the test product induced no delayed irritation responses.

**Table 5:** Molecular improvements based on gene expression changes after 2 and 4 weeks of topical application in participants with sensitive skin.

		*MCOLN1*	*CYR61*	*PMAIP1*	*PTGS2*	*HMGB2*
No. of Analyzed Subjects		22	23	23	21	21
Mean ± SD	Before	16.883 ± 2.215	1.395 ± 1.145	−2.551 ± 3.766	11.259 ± 3.049	1.722 ± 3.739
	After 2 weeks	17.987 ± 1.916	2.266 ± 1.288	4.460 ± 6.044	12.656 ± 3.699	4.459 ± 3.642
	After 4 weeks	18.665 ± 2.221	1.938 ± 2.208	3.235 ± 6.024	14.015 ± 4.921	3.633 ± 5.356
Rate of change (%)	Before–after 2 weeks	6.542	62.438	274.796	12.405	158.933
	Before–after 4 weeks	10.554	38.965	226.806	24.476	111.007
No. of improved subjects (*n*/%)	Before–after 2 weeks	14/63.636	18/78.261	22/95.652	15/71.429	19/90.476
	Before–after 4 weeks	17/77.273	12/52.174	21/91.304	14/66.667	18/85.714
Statistical analysis (*p*-value)	Between-effect model	0.005 *	0.054	<0.001 *	0.064	<0.001 *
	Before–after 2 weeks	0.114	0.016 *	<0.001 *	0.224	0.004 *
	Before–after 4 weeks	0.010 *	0.330	<0.001 *	0.078	0.005 *

* Rate of Change (%) = ((Mean After C_T_ − Mean Before C_T_)/ABS(Mean Before C_T_)) × 100.

**Table 6:** Physiological improvements after 2 and 4 weeks of topical application in participants with sensitive skin.

		Skin Redness (a*)	TEWL (g/m^2^/h)	Skin Moisture (ε)	LAST Score
Mean ± SD	Before	8.713 ± 2.352	16.275 ± 4.498	67.217 ± 7.090	0.518 ± 0.266
	After 2 weeks	8.360 ± 2.216	14.482 ± 5.015	75.832 ± 6.836	0.257 ± 0.178
	After 4 weeks	8.102 ± 2.110	14.121 ± 5.066	75.038 ± 10.604	0.137 ± 0.206
Rate of change (%)	Before–after 2 weeks	4.052	11.021	12.816	50.294
	Before–after 4 weeks	7.011	13.238	11.634	73.468
No. of improved subjects (*n*/%)	Before–after 2 weeks	15/65.217	18/78.261	23/100.000	16/69.565
	Before–after 4 weeks	16/69.565	18/78.261	22/95.652	22/95.652
Statistical analysis (*p*-value)	Between-effect model	0.032 *	0.004 *	<0.001 *	<0.001 *
	Before–after 2 weeks	0.339	0.009 *	<0.001 *	0.002 *
	Before–after 4 weeks	0.049 *	0.018 *	<0.001 *	<0.001 *

* Rate of Change (%) = ((Mean After − Mean Before)/ABS(Mean Before)) × 100.

**Table 7:** Summary of biomarker-based cosmetic ingredient discovery and validation.

Biomarker	Known Role in Sensitive Skin	Matched Cosmetic Ingredient	In Vitro Efficacy	Clinical Efficacy *
*MCOLN1*	Ca^2+^ transport via TRP channels	Schizandrin B (Schisandra)	Significant	Significant (week 4)
*CYR61*	Inflammatory response mediator	Genistein	Significant	Significant (week 2)
*PMAIP1*	Apoptotic signaling	Folic acid	Significant	Significant (weeks 2 and 4)
*PTGS2*	Prostaglandin synthesis	Allantoin	Significant	Not significant (tendency)
*HMGB2*	Inflammatory signal transduction	Betaine	Significant	Significant (week 2)

* Clinical efficacy was assessed using a prototype emulsion containing all five ingredients. In vitro and clinical efficacy was evaluated via qRT-PCR-based biomarker expression analysis.

## Data Availability

The microarray data used in this study have been deposited in the NCBI Gene Expression Omnibus (GEO) database (accession number GSE179925). The dataset is currently restricted and will be publicly available from July 2028. Data are available from the corresponding author upon reasonable request prior to public release.

## Supplementary Materials

The following supporting information can be downloaded at https://www.mdpi.com/article/10.3390/biom16060843/s1. Figure S1: Cytotoxicity assessment of cosmetic ingredients in HaCaT cells; Figure S2: Cytotoxicity assessment of cosmetic ingredients in human dermal fibroblasts (HDFs); Table S1: Demographic characteristics of study participants enrolled in the 4-week open-label clinical efficacy trial.

## Abbreviations

The following abbreviations are used in this manuscript:

MISSM	Minimally Invasive Skin Sampling Method using Microneedle Patches
PCA	Principal Component Analysis
GSEA	Gene Set Enrichment Analysis
GO_BP	Gene Ontology Biological Process
KEGG	Kyoto Encyclopedia of Genes and Genomes
OD	Optical Density
DMEM	Dulbecco’s modified Eagle’s medium
HDFs	Human dermal fibroblasts
MTT	3-(4,5-dimethylthiazol-2-yl)-2,5-diphenyltetrazolium bromide
TEWL	Transepidermal Water Loss
LAST	Lactic Acid Stinging Test
C_T_	Cycle Threshold
ANOVA	Analysis of Variance
PCR	Polymerase Chain Reaction
*MCOLN1*	Mucolipin-1
*CYR61*	Cysteine Rich Angiogenic Inducer 61
*PMAIP1*	Phorbol-12-Myristate-13-Acetate-Induced Protein 1
*PTGS2*	Prostaglandin-Endoperoxide Synthase 2
*HMGB2*	High-Mobility Group Box 2

## References

[1] Berardesca E., Farage M., Maibach H. (2013). Sensitive skin: An overview. Int. J. Cosmet. Sci..

[2] Brenaut E., Barnetche T., Le Gall-Ianotto C., Roudot A.C., Misery L., Ficheux A.S. (2020). Triggering factors in sensitive skin from the worldwide patients’ point of view: A systematic literature review and meta-analysis. J. Eur. Acad. Dermatol. Venereol..

[3] Misery L., Ständer S., Szepietowski J.C., Reich A., Wallengren J., Evers A.W., Takamori K., Brenaut E., Le Gall-Ianotto C., Fluhr J. (2017). Definition of Sensitive Skin: An Expert Position Paper from the Special Interest Group on Sensitive Skin of the International Forum for the Study of Itch. Acta Derm. Venereol..

[4] Do L.H.D., Azizi N., Maibach H. (2020). Sensitive Skin Syndrome: An Update. Am. J. Clin. Dermatol..

[5] Misery L., Boussetta S., Nocera T., Perez-Cullell N., Taieb C. (2009). Sensitive skin in Europe. J. Eur. Acad. Dermatol. Venereol..

[6] Willis C.M., Shaw S., De Lacharrière O., Baverel M., Reiche L., Jourdain R., Bastien P., Wilkinson J.D. (2001). Sensitive skin: An epidemiological study. Br. J. Dermatol..

[7] Farage M.A., Maibach H.I. (2010). Sensitive skin: Closing in on a physiological cause. Contact Dermat..

[8] Bialas I., Zelent-Kraciuk S., Jurowski K. (2023). The Skin Sensitisation of Cosmetic Ingredients: Review of Actual Regulatory Status. Toxics.

[9] Kim E.J., Quan Q.L., Cho S.I., Kim Y.K., Lee D.H., Chung J.H. (2024). The novel adiponectin receptor agonist APN5N alleviates sensitive skin by upregulating adiponectin expression. J. Dermatol. Sci..

[10] Xiao T., Sun M., Zhao C., Kang J. (2023). TRPV1: A promising therapeutic target for skin aging and inflammatory skin diseases. Front. Pharmacol..

[11] Fan L., He C., Jiang L., Bi Y., Dong Y., Jia Y. (2016). Brief analysis of causes of sensitive skin and advances in evaluation of anti-allergic activity of cosmetic products. Int. J. Cosmet. Sci..

[12] Roussaki-Schulze A.V., Zafiriou E., Nikoulis D., Klimi E., Rallis E., Zintzaras E. (2005). Objective biophysical findings in patients with sensitive skin. Drugs Exp. Clin. Res..

[13] Kim S.H., Kim J.H., Lee S.J., Jung M.S., Jeong D.H., Lee K.H. (2022). Minimally invasive skin sampling and transcriptome analysis using microneedles for skin type biomarker research. Ski. Res. Technol..

[14] Wang H., Huang Z., Lou S., Li W., Liu G., Tang Y. (2024). In Silico Prediction of Skin Sensitization for Compounds via Flexible Evidence Combination Based on Machine Learning and Dempster-Shafer Theory. Chem. Res. Toxicol..

[15] Ta G.H., Weng C.F., Leong M.K. (2021). In silico Prediction of Skin Sensitization: Quo vadis? *Front*. Pharmacol..

[16] Yeh S.J., Lin J.F., Chen B.S. (2021). Multiple-Molecule Drug Design Based on Systems Biology Approaches and Deep Neural Network to Mitigate Human Skin Aging. Molecules.

[17] Jasly K., Goyal S., Ashwini P.K., Kanthraj G.R., Chethana S.G., Ranugha S. (2024). Three-part scoring system (tripartite) for teledermatology versus International Contact Dermatitis Research Group criteria to interpret patch test readings: A comparative, observational study. Indian J. Dermatol. Venereol. Leprol..

[18] Marriott M., Holmes J., Peters L., Cooper K., Rowson M., Basketter D.A. (2005). The complex problem of sensitive skin. Contact Dermat..

[19] Kim S.H., Kim J.H., Choi Y.M., Seo S.M., Jang E.Y., Lee S.J., Zhang H.S., Roh Y., Jung Y.W., Park C.O. (2024). Development of a biomarker-based platform for comprehensive skin characterization using minimally invasive skin sampling and quantitative real-time PCR. Ski. Res. Technol..

[20] Zhang X., Cheng X., Yu L., Yang J., Calvo R., Patnaik S., Hu X., Gao Q., Yang M., Lawas M. (2016). *MCOLN1* is a ROS sensor in lysosomes that regulates autophagy. Nat. Commun..

[21] Zhang H., Chen Q., Dahan A., Xue J., Wei L., Tan W., Zhang G. (2019). Transcriptomic analyses reveal the molecular mechanisms of schisandrin B alleviates CCl_4_-induced liver fibrosis in rats by RNA-sequencing. Chem. Biol. Interact..

[22] Di X., Andrews D.M., Tucker C.J., Yu L., Moore A.B., Zheng X., Castro L., Hermon T., Xiao H., Dixon D. (2012). A high concentration of genistein down-regulates activin A, Smad3 and other TGF-β pathway genes in human uterine leiomyoma cells. Exp. Mol. Med..

[23] Sotoca A.M., Gelpke M.D.S., Boeren S., Ström A., Gustafsson J., Murk A.J., Rietjens I.M.C.M., Vervoort J. (2011). Quantitative proteomics and transcriptomics addressing the estrogen receptor subtype-mediated effects in T47D breast cancer cells exposed to the phytoestrogen genistein. Mol. Cell. Proteom..

[24] Barua S., Kuizon S., Chadman K.K., Brown W.T., Junaid M.A. (2015). Microarray analysis reveals higher gestational folic Acid alters expression of genes in the cerebellum of mice offspring—A pilot study. Brain Sci..

[25] Tan W.S., Arulselvan P., Ng S.F., Mat Taib C.N., Sarian M.N., Fakurazi S. (2019). Improvement of diabetic wound healing by topical application of Vicenin-2 hydrocolloid film on Sprague Dawley rats. BMC Complement. Altern. Med..

[26] Rauhala L., Hämäläinen L., Dunlop T.W., Pehkonen P., Bart G., Kokkonen M., Tammi M., Tammi R., Pasonen-Seppänen S. (2015). The organic osmolyte betaine induces keratin 2 expression in rat epidermal keratinocytes—A genome-wide study in UVB irradiated organotypic 3D cultures. Toxicol. Vitr..

[27] Baumann L. (2008). Understanding and treating various skin types: The Baumann Skin Type Indicator. Dermatol. Clin..

[28] Draelos Z.D. (1997). Sensitive skin: Perceptions, evaluation, and treatment. Am. J. Contact Dermat..

[29] Chan K.T.M. (2018). Sensitive Skin Syndromes and Transient Receptors Potential (TRP) Channels in Sensitive Skin. Int. J. Clin. Exp. Dermatol..

[30] Kim S.H., Kim J.H., Choi Y.M., Seo S.M., Jang E.Y., Lee S.J., Cho S., Jeong D.H., Lee K.H. (2024). Microneedles: A novel clinical technology for evaluating skin characteristics. Ski. Res. Technol..

[31] Qian F., Noben-Trauth K. (2005). Cellular and molecular function of mucolipins (TRPML) and polycystin 2 (TRPP2). Pflügers Arch..

[32] Medina D.L., Di Paola S., Peluso I., Armani A., De Stefani D., Venditti R., Montefusco S., Scotto-Rosato A., Prezioso C., Forrester A. (2015). Lysosomal calcium signalling regulates autophagy through calcineurin and TFEB. Nat. Cell Biol..

[33] Pitake S., Middleton L.J., Abdus-Saboor I., Mishra S.K. (2019). Inflammation Induced Sensory Nerve Growth and Pain Hypersensitivity Requires the N-Type Calcium Channel Cav2.2. Front. Neurosci..

[34] Chelombitko M.A., Chernyak B.V., Fedorov A.V., Zinovkin R.A., Razin E., Paruchuru L.B. (2020). The Role Played by Mitochondria in FcεRI-Dependent Mast Cell Activation. Front. Immunol..

[35] Pavlyuchenkova A.N., Smirnov M.S., Chernyak B.V., Chelombitko M.A. (2024). The Role Played by Autophagy in FcεRI-Dependent Activation of Mast Cells. Cells.

[36] Sun Y., Zhang J., Zhai T., Li H., Li H., Huo R., Shen B., Wang B., Chen X., Li N. (2017). CCN1 promotes IL-1β production in keratinocytes by activating p38 MAPK signaling in psoriasis. Sci. Rep..

[37] Fahey E., Doyle S.L. (2019). IL-1 Family Cytokine Regulation of Vascular Permeability and Angiogenesis. Front. Immunol..

[38] Chen N., Chen C.C., Lau L.F. (2000). Adhesion of human skin fibroblasts to *Cyr61* is mediated through integrin α_6_β_1_ and cell surface heparan sulfate proteoglycans. J. Biol. Chem..

[39] Chen N., Leu S.J., Todorovic V., Lam S.C.-T., Lau L.F. (2004). Identification of a novel integrin α_v_β_3_ binding site in CCN1 (*CYR61*) critical for pro-angiogenic activities in vascular endothelial cells. J. Biol. Chem..

[40] Naik E., Michalak E.M., Villunger A., Adams J.M., Strasser A. (2007). Ultraviolet radiation triggers apoptosis of fibroblasts and skin keratinocytes mainly via the BH3-only protein Noxa. J. Cell Biol..

[41] Fujii N., Singh M.S., Halili L., Boulay P., Sigal R.J., Kenny G.P. (2016). Cutaneous vascular and sweating responses to intradermal administration of prostaglandin E1 and E2 in young and older adults: A role for nitric oxide?. Am. J. Physiol.-Regul. Integr. Comp. Physiol..

[42] Medow M.S., Glover J.L., Stewart J.M. (2008). Nitric oxide and prostaglandin inhibition during acetylcholine-mediated cutaneous vasodilation in humans. Microcirculation.

[43] Niu L., Yang W., Duan L., Wang X., Li Y., Xu C., Liu C., Zhang Y., Zhou W., Liu J. (2020). Biological functions and theranostic potential of HMGB family members in human cancers. Ther. Adv. Med. Oncol..

[44] Qu W.F., Zhu G.Q., Yang R., Chu T.H., Guan Z.Q., Huang R., Tian M.X., Jiang X.F., Tao C.Y., Fang Y. (2025). Targeting *HMGB2* acts as dual immunomodulator by bolstering CD8^+^ T cell function and inhibiting tumor growth in hepatocellular carcinoma. Sci. Adv..

