# Supplementary material for: Sensitive Skin Improvement Through Bioinformatics-Identified Cosmetic Ingredients That Regulate Transcriptome-Derived Biomarkers
Source: Biomolecules. 2026 Jun 9;16(6):843. doi: 10.3390/biom16060843 (PMC13297263; doi:10.3390/biom16060843)
Supplement: Supplementary file 1 [file biomolecules-16-00843-s001.zip › biomolecules-4318808-supplementary.pdf]

Supplementary Table and Figures

Table S1. Demographic Characteristics of Study Participants

	**Subjects**
Number (N)	23
Sex	
Male	0
Female	23
Age (yr)	47.26 ± 6.81

Summary of age and sex distribution of the 23 participants enrolled in the 4-week open-label clinical efficacy trial for sensitive skin.

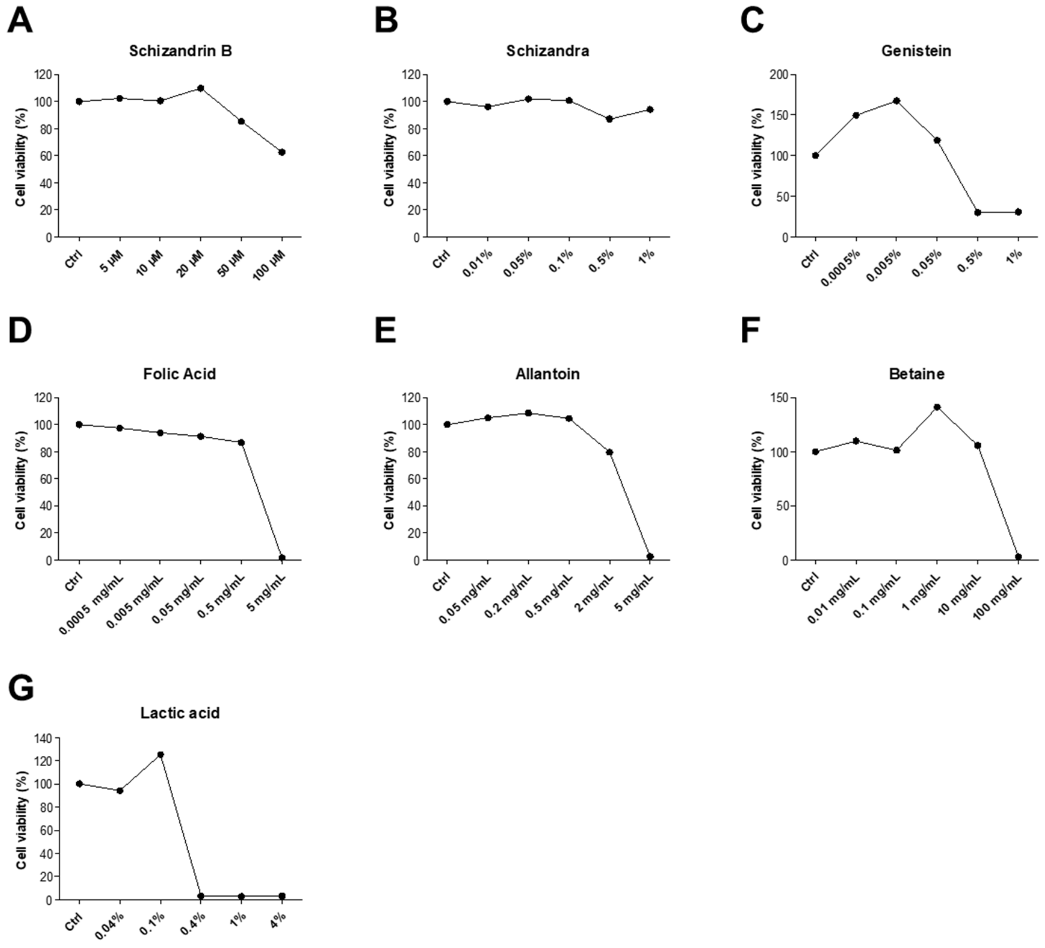

Figure S1. Cytotoxicity assessment of cosmetic ingredients in HaCaT cells

MTT assay was performed to evaluate dose-dependent cytotoxicity of (**A–F**) candidate ingredients and (**G**) lactic acid in HaCaT cells. Cells were exposed to serial dilutions of each compound for 24 h, and cell viability was calculated. Non-cytotoxic concentrations were selected for further experiments.

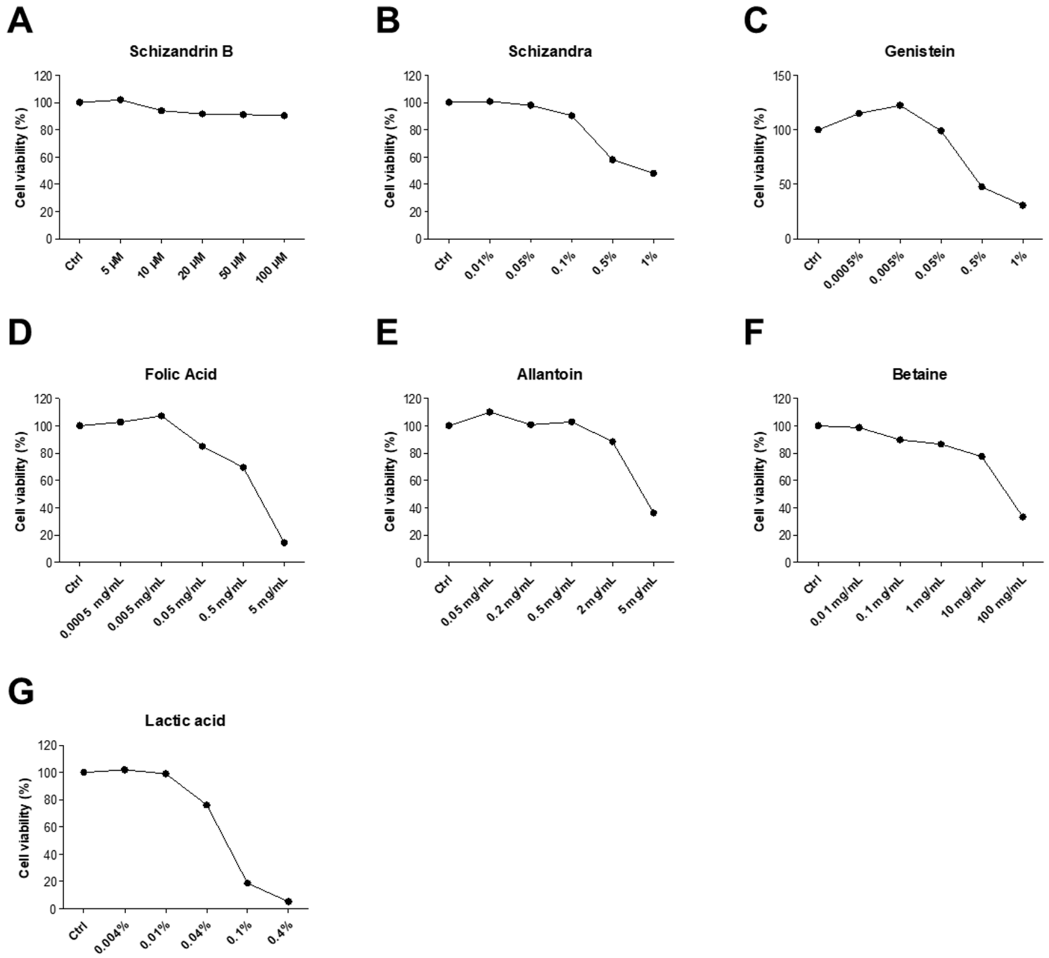

Figure S2. Cytotoxicity assessment of cosmetic ingredients in human dermal fibroblasts (HDFs)

MTT assay was performed to evaluate dose-dependent cytotoxicity of (**A–F**) candidate ingredients and (**G**) lactic acid in HDF cells. Cells were exposed to serial dilutions of each compound for 24 h, and cell viability was calculated. Non-cytotoxic concentrations were selected for further experiments.
